# Single and Combined Effect of Mild-Heat Treatment and Alginate Coatings on Quality Preservation of Minimally Processed Bunching Green Onions

**DOI:** 10.3390/foods11050641

**Published:** 2022-02-23

**Authors:** Carolina Medina-Jaramillo, Karen Usgame-Fagua, Nelson Franco-González, Alex López-Córdoba

**Affiliations:** Grupo de Investigación en Bioeconomía y Sostenibilidad Agroalimentaria, Escuela de Administración de Empresas Agropecuarias, Facultad Seccional Duitama, Universidad Pedagógica y Tecnológica de Colombia, Carrera 18 con Calle 22, Duitama 150461, Colombia; caromedina1986@gmail.com (C.M.-J.); karen.usgame@uptc.edu.co (K.U.-F.); nelson.franco@uptc.edu.co (N.F.-G.)

**Keywords:** food loss and waste, food preservation, hurdle technology, minimal processing

## Abstract

Bunching green onion is an *Allium* species that has been widely used in food flavorings and seasonings. This vegetable experiences a rapid loss of quality during storage due to physiological changes and microbial spoilage. In the current work, the single and combined effect of mild-heat treatment (55 °C for 60 s) and alginate edible coatings on the quality preservation of minimally processed bunching green onions was studied. Control and treated samples were stored at 4 °C for 15 days and examined periodically in terms of their respiration rate, weight loss, pH, soluble solids content, firmness, total polyphenol content, antioxidant activity, microbial count, decay ratio, and overall visual quality. The results showed that the combination of mild heat and alginate edible coatings was the most effective approach to slow down the respiration rate and the incidence of decay in the minimally processed bunching green onions. In addition, the treatments with alginate coating alone or combined with mild-heat treatment showed the best performance for maintaining the overall visual quality of the products during the storage.

## 1. Introduction

As a result of changing consumer habits, the global market for minimally processed fruits and vegetables (MPFVs) has developed rapidly in recent years [1]. It is estimated that this market will reach USD 346.05 billion by 2022 [2,3]. Some of the minimally processed products in the market are the mixtures of vegetables to salads, soups, and sandwiches [4]. 

Bunching green onion (also called scallion, green onion, Welsh onion, salad onion, and Japanese bunching onion) is an *Allium* species known for its distinctive flavor, aroma, and pungency [5]. In addition, this vegetable is widely recognized for its use in food flavorings and seasonings and its therapeutic role because of its antioxidant, anti-inflammatory, and hypo-cholesterolemic properties [6,7]. As with most fruits and vegetables, bunching green onion provides a major challenge as a minimally processed product, as it rapidly undergoes physiological and biochemical changes that affect its shelf life, such as weight loss, softening, color changes, and microbial and enzymatic spoilage [5,8]. It is, therefore, necessary to develop strategies to extend the marketability of minimally processed bunching green onions. 

To date, some preservation approaches have been used to retain the quality of fresh and minimally processed bunching green onions, including refrigerated storage, controlled atmosphere, mild-heat treatments, chemical preservatives (e.g., 1-MCP, peroxyacetic acid, and chloride), ultrasound, and edible coatings [5,9,10,11,12,13,14]. 

Mild-heat treatment (MHT) is a physical preservation method that is usually applied by immersion in water at temperatures ranging from 30 to 60 °C, for periods of up to 20 min. Several authors have reported that this treatment slows down the senescence, inhibits the development of physiological disorders, and reduces microbial population in MPFVs [1,15,16,17,18,19]. In particular, it has been reported that the application of mild-heat treatments delays the browning and reduces the extension grown and the mesophilic aerobic population of minimally processed bunching green onions [9,12].

Edible coatings have also been evaluated as an alternative to preserving the quality of a wide variety of MPFVs [4,20,21]. These are generally made based on blends of film-forming edible polymers (e.g., starch, pectin, and alginate), solvents (e.g., water and ethanol), as well as other additives (e.g., plasticizers, surfactants, emulsifiers, natural extracts and essential oils, among others) [4,20,21]. Several studies have reported that the application of edible coatings in MPFVs decrease respiration rate, reduce weight and aroma loss, delay color changes, improve gloss and firmness, and reduce microbial growth [4,20,21,22].

Alginates are polysaccharides composed of β-d-mannuronic acid and α-l-guluronic acid monomers linked by 1–4 glycosidic bonds, which are widely used in the fabrication of edible coatings for MPFVs due to their good film-forming properties and oxygen barrier properties [20,23,24,25,26,27]. In addition, alginates are odorless, tasteless, and colorless so they do not affect the food sensory quality [22,28]. Alginate edible coatings alone or incorporated with active agents (e.g., essential oils and herbal extracts) have been applied to several MPFVs, including pineapples [29], mangoes [30], melons [31], and apples [32]. However, there is little research about the use of alginate coatings on minimally processed bunching green onion. Rozo et al. reported that the application of calcium alginate edible coatings on bunching green onions decreased the weight loss and delayed the pH changes during storage [11].

The combination of different preservation methods is also considered a useful strategy to prevent the spoilage of MPFVs. It has been reported that the combined application of preservation methods could enhance the microbial safety of these products [19]. Moreira et al. studied the efficacy of the application of mild-heat treatments combined with either chitosan or carboxymethylcellulose edible coatings for controlling microbiological deterioration and maintaining the sensory attributes of fresh-cut broccoli [18,33]. The findings indicated that the application of thermal shocks prior to chitosan coating diminished the weight loss and the microbial count during storage. Koh et al. stated that the combined application of alginate coating and repetitive pulsed light reduced the microbial growth of fresh-cut cantaloupe [34]. Ben-Fadhel et al. [35] observed that the application of calcium caseinate edible coatings combined with γ-irradiation exhibited a synergistic potential and a better efficiency in extending the shelf-life of fresh-cut carrots.

At present, no studies are available regarding the effect of the application of mild-heat treatments combined with alginate edible coatings on minimally processed bunching green onion. The aim of the current work was to evaluate the effect of mild-heat treatment (55 °C for 60 s), alginate coatings, and their combination on quality preservation of minimally processed bunching green onions stored at 4 °C for 15 days. Control and treated samples were evaluated periodically in terms of their physicochemical, microbiological, and sensory attributes to define the most suitable preservation method for the minimally processed product.

## 2. Materials and Methods

### 2.1. Materials

Fresh bunching green onions (*Allium fistulosum*) were obtained in a local market of Duitama city (Boyacá, Colombia) and held at 4 °C and 90% RH until use (within 24 h). Before being used, the bulbs were examined to identify those with physical, mechanical, or microbiological damage. 

Sodium alginate was generously provided by Saporiti (Buenos Aires, Argentina). Glycerol was supplied by J. T. Baker (Phillipsburg, NJ, USA). Sodium hydroxide and calcium chloride were supplied by Sigma-Aldrich (St. Louis, MO, USA). Folin-Ciocalteu reagent was provided by Panreac (Barcelona, Spain), and gallic acid was provided by Merck (Darmstadt, Germany). All other reagents used were of analytical grade.

### 2.2. Minimal Processing

The minimal processing was performed following the methodology described by Hong et al. and Zudaire et al. [9,14]. Briefly, bunching green onions were washed with a 100 mg L^−1^ NaClO solution, peeled, trimmed (roots and leaf tips cut), and rinsed with water. Finally, trimmed stalks were crosscut into 10-cm lengths.

### 2.3. Coating-Forming Formulations

Coating solutions were prepared following the protocol described by Medina-Jaramillo et al. [26]. Briefly, sodium alginate powder (2 g/100 mL) was dissolved in distilled water heated to 70 °C with steady stirring until the blend turned transparent.

After cooling, glycerol (30 g/100 g dry solids) was added to the sodium alginate solution as a plasticizer and stirred for 10 min. To aid dispersion, Tween 20 (5 g/100 g dry solids) was added to the alginate/glycerol blends.

All coating solutions were blended at 5000 rpm for 3 min in an Ultra Turrax T25 homogenizer (IKA^®^ WERKE, Staufen, Germany), degassed, and cooled to room temperature before applying to the minimally processed bunching green onions.

### 2.4. Mild-Heat Treatments and Coating Application 

A total of 8.4 kg of minimally processed bunching green onions were randomly distributed into four groups, each group containing 2.1 kg of sample: (1) Control: minimally processed bunching green onions were immersed in distilled water and air-dried at room temperature. (2) Mild-heat treatment (MHT): minimally processed onions were immersed in water at 55 °C for 60 s. (3) Edible coating (EC) application: the samples were coated by brushing the alginate coating solutions on onion faces and allowing them to dry for 3 min at room temperature (15–20 °C). Once the onion surface had dried fully, two successive coatings were applied. Finally, the samples were immersed in a calcium chloride solution (2 g/100 mL) for 30 min, rinsed with distilled water, and air-dried at room temperature [26,28]. (4) Combination of mild-heat treatment and coating applications (MHT + EC): the samples were subjected to MHT followed by alginate edible coating application.

### 2.5. Quality Evaluation of Minimally Processed Onions during Storage

Control and treated minimally processed bunching green onions were put in polyethylene terephthalate (PET) packages and stored for 15 days at 4 °C and 90% RH. Quality attributes were evaluated at 0, 3, 6, 9, and 15 days of assay. Three packages containing 140 g of minimally processed onions were produced for each sampling time.

#### 2.5.1. Respiration Rate 

Respiration rate was determined using a closed system as previously described by Medina-Jaramillo et al. [28]. Approximately 140 g of sample was placed within hermetically sealed 2 L flasks for 30 min at 25 °C. After that, an infrared analyzer (LabQuest^®^ 2 Model LQ2-LE, Beaverton, OR, USA) was used to determine the carbon dioxide (CO_2_) amount. The respiration rate was expressed in mg CO_2_ kg^−1^ s^−1^. 

#### 2.5.2. Weight Loss

The weight loss (% *W*) was determined at each sampling time as a percentage of variation regarding the initial weight according to the following equation:

(1) $$\%\;W=\left(\frac{m_{0}-m_{f}}{m_{0}}\right)\times 100,$$

where *m*_f_ is the weight at each time and *m*_0_ is the sample’s initial weight.

#### 2.5.3. pH, Soluble Solids Content, and Firmness

A digital pH meter (Oakton Instruments, Vernon Hills, IL, USA) was used to determine the pH of the samples (AOAC 981.12). 

The soluble solids content (°Brix) was determined in the onion juice using a refractometer (model PR 101, Atago Co., Tokyo, Japan) (AOAC 932.12). 

Firmness was determined using a digital Force Gauge PCE-FM200 (Southampton, UK) with a 6 mm diameter stainless steel probe. 

#### 2.5.4. Total Polyphenol Content and Antioxidant Activity

The total polyphenol content and the antioxidant activity of the samples were evaluated using the methods described in our previous works [28,36]. The ground samples (~1.7 g) were introduced in tubes and blended with ethanol at 70% *v*/*v* (3 mL). Then, the tubes with the blends were immersed in water at 60 °C in the ultrasonic bath (Branson CPX1800, Danbury, CT, USA) for 10 min. After that, the obtained extracts were cooled and filtered. 

The Folin–Ciocalteu method was used to determine the total polyphenols content [37,38]. The results were expressed as gallic acid equivalents (GAE) per gram of wet sample.

Antioxidant activity was measured by the Brand-Williams method [39]. The results were expressed as gallic acid equivalents (GAE) per gram of wet sample.

#### 2.5.5. Microbiological Analysis 

Microbial counts were performed following the protocol described in a previous work [28]. The determination of aerobic mesophilic bacteria was done following ISO 4833-1: 2013 standard [40]. To count molds and yeasts, the assay was performed according to ISO 21527-1,2: 2008 standard [41]. Colonies were enumerated and the results were reported as log colony-forming units per gram (log CFU · g^−1^).

#### 2.5.6. Decay Ratio

The surface appearance of the samples was visually examined at each sampling time. Samples that showed soft rot, discoloration defects, or fungal growth were considered decayed [14]. Three different assays were carried out, each in triplicate. Results were expressed as the ratio between the amount of the decayed samples to the total amount of samples.

#### 2.5.7. Consumer Assessment

Control and treated minimally processed onions were presented to an untrained panel of sixty-six consumers, ranging in age from 18 to 60 years old, at the initial time and at each sampling day (3, 6, 9, and 15 days) [42]. The total number of participating consumers was 330, i.e., 66 consumers for each sampling date. 

The samples were placed in polyethylene terephthalate (PET) trays labeled with 3-digit random numbers. The consumers were asked to evaluate the overall visual quality on a 9-point hedonic scale (1 = dislike extremely, 5 = neither like nor dislike, and 9 = like extremely).

### 2.6. Statistical Analysis 

Minitab v. 16 statistical software (Minitab Inc., State College, PA, USA) was used for the statistical analysis. Analysis of variance and Tukey’s pairwise comparisons were performed with a 95% confidence level. The studies were done at least in triplicate, and the results were given as mean ± standard deviation.

## 3. Results and Discussion

### 3.1. Respiration Rate and Weight Loss

Respiration is one of the most important physiological processes associated with the ripening, senescence, and deterioration of horticultural products after harvest [19]. In general, fruits and vegetables with a high respiration rate are often perishable [19]. 

**Figure 1:**
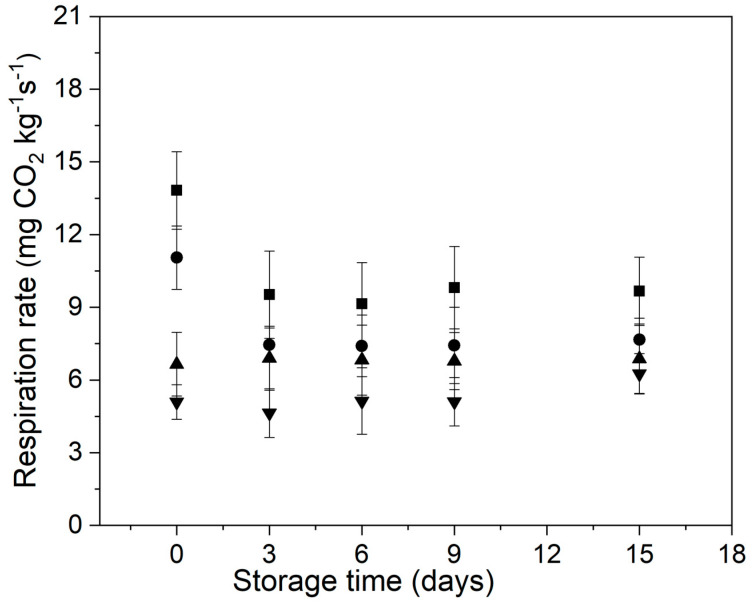
Behavior of the respiration rate of control and treated minimally processed bunching green onions during storage. Control (■); mild-heat treatment (●); edible coating (▲); mild-heat treatment+ edible coating (▼).

Figure 1 shows the behavior of the respiration rate of control and treated minimally processed bunching green onion during storage. Overall, control samples showed a higher respiration rate than the treated ones during the entire storage. In addition, the samples MHT + EC exhibited the lower CO_2_ production between all samples, during the first 9 days of assay. 

The respiration rate of control and MHT samples decreased during the first 3 days of storage but was subsequently maintained until day 15 of assay, while in the samples EC and MHT + EC this parameter barely changed during the entire storage. 

Several authors have reported that the carbohydrate-based edible coatings (e.g., alginate and chitosan) affect the vegetable surface, generating an internal atmosphere of relatively high carbon dioxide and low oxygen, which slows down the respiration rate and thus causes a reduction of the intensity of physiological processes [43,44,45].

Water loss is dependent on several factors, including the relative humidity of the surrounding atmosphere and the external surface of the product [19,46]. Some studies have reported that mild-heat treatments and edible coatings could be useful hurdles to prevent water loss in MPFVs [33,46]. 

**Figure 2:**
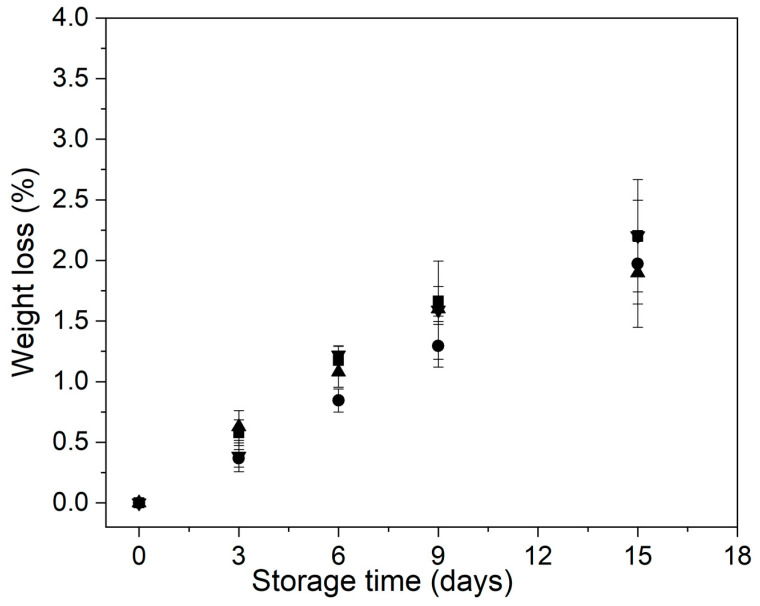
Behavior of the weight loss of control and treated minimally processed bunching green onion during storage. Control (■); mild-heat treatment (●); edible coating (▲); mild-heat treatment+ edible coating (▼).

The behavior of the weight loss of control and treated minimally processed bunching green onion during storage is shown in Figure 2. The different treatments applied did not cause significant changes in the weight loss of the minimally processed bunching green onions (*p* > 0.05). Control and treated samples showed a progressive increase in the percentage of weight loss, reaching values ranging between 1.8 and 2.2%, at day 15 of storage (Figure 2). Zudaire et al. [9] and Lwin et al. [5] reported a higher weight loss for calçots stored at 4 °C for 15 days (4.8%) and bunching onions held at 10 °C for 10 days (10%), respectively. 

On the other hand, Siddiq et al. reported that the application of mild-heat treatments beyond 50 °C affected the tissue structure of fresh-cut onions, causing shrinkage and thus an increase in the weight loss [47]. Therefore, it can be suggested that the applied treatments were suitable so as not to increase the water migration from the minimally processed bunching green onions. 

### 3.2. pH, Total Soluble Solids Content and Firmness

**Figure 3:**
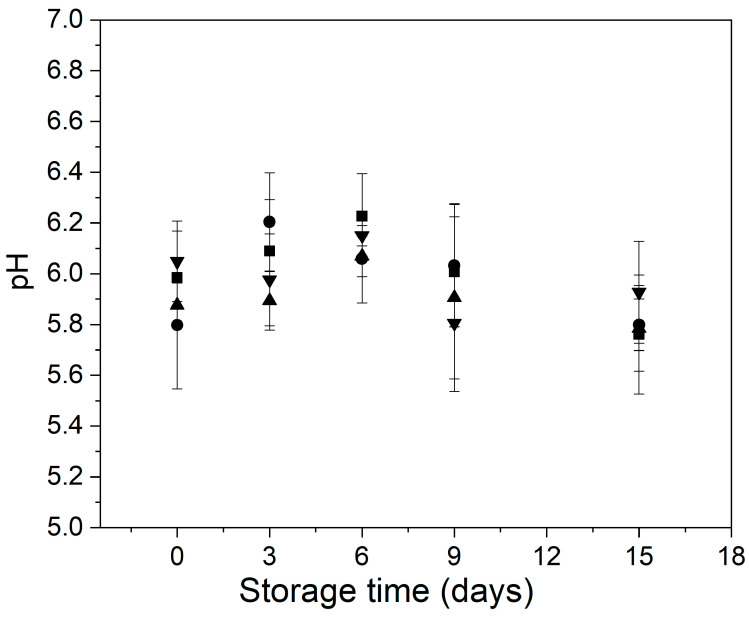
Behavior of the pH of control and treated minimally processed bunching green onions during storage. Control (■); mild-heat treatment (●); edible coating (▲); mild-heat treatment+ edible coating (▼).

Figure 3 shows the behavior of the pH of control and treated minimally processed bunching green onions during storage. Overall, the different treatments applied did not cause significant changes in the pH of the minimally processed bunching green onions (Figure 3). At the initial time, all samples showed values of pH ranging between 5.8 and 6.0, characteristics of low acid foods which are susceptible to microbial proliferation [48]. Other authors have reported similar pH values for green onions of several varieties [11,49]. 

At the end of the storage, control and treated samples showed similar pH values as at the initial time (Figure 3). From the preservation point of view, it is positive because changes in pH could promote color and texture loss [50,51]. Zudaire et al. [49] observed a decrease in the pH of fresh-cut calçots held in a controlled atmosphere for 15 days at 4 °C.

**Figure 4:**
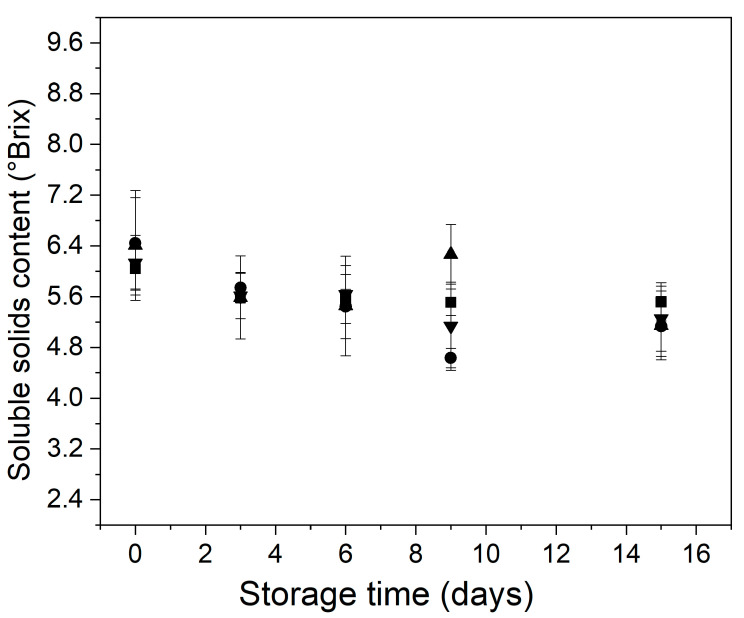
Behavior of the soluble solids content of control and treated minimally processed bunching green onion during storage. Control (■); mild-heat treatment (●); edible coating (▲); mild-heat treatment+ edible coating (▼).

The behavior of the soluble solids content of control and treated minimally processed bunching green onion during storage is shown in Figure 4. It has been reported that the application of edible coatings delayed the changes in the soluble solids content of fresh and minimally processed fruits and vegetables [52,53].

In the current work, the single or combined application of mild-heat treatments and alginate edible coatings did not generate significant changes in the soluble solids content of minimally processed bunching green onion. At the initial time, control and treated samples showed a soluble solids content raging between 6.0 and 6.5 °Brix. Then, the soluble solids content of the samples decreased slowly until the end of the storage, and no significant differences (*p* > 0.05) were observed among them at day 15 of the assay (Figure 4). These results were like those reported by Han et al. for fresh-cut Welsh onion, where the soluble solids content decreased after 7 days of storage at 4 °C [54]. 

**Figure 5:**
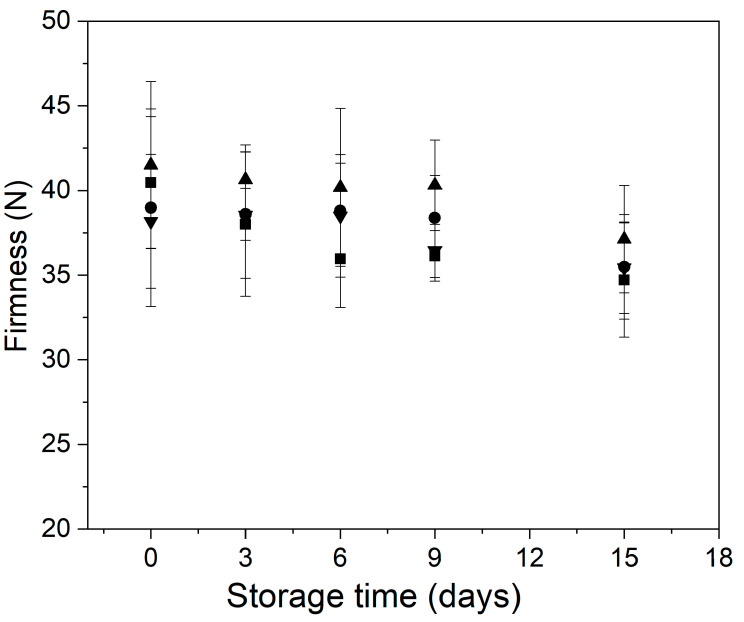
Behavior of the firmness of control and treated minimally processed bunching green onion during storage. Control (■); mild-heat treatment (●); edible coating (▲); mild-heat treatment+ edible coating (▼).

Figure 5 shows the behavior of the firmness of control and treated minimally processed bunching onion during storage. Overall, the samples with the single application of edible coating showed higher firmness than the samples control and with thermal treatment (MHT and MHT + EC) during storage. At the initial time, it was observed that the samples with thermal treatment (MHT and MHT + EC) showed a slight decrease in the firmness concerning the control and EC samples. It has been reported that the application of thermal treatment could affect the tissue structure of minimally processed vegetables, causing softening [33]. After day 3 of storage, control samples exhibited a significant decrease in firmness, whereas in the samples MHT + EC the firmness decreased after 6 days of the assay. In the case of the samples with a single application of edible coating (EC) or mild-heat treatment (MHT), the softening was delayed and the minimally processed bunching green onions preserved their firmness from beginning of storage until day 9. These findings agree with the fact that the treated samples had a lower respiration rate (Figure 1). 

At the end of the storage, all samples showed a decrease in the firmness concerning the initial time. This behavior was probably due to the softening generated by the increase in their metabolic and enzymatic activity [8]. 

### 3.3. Total Polyphenol Content and Antioxidant Activity 

**Figure 6:**
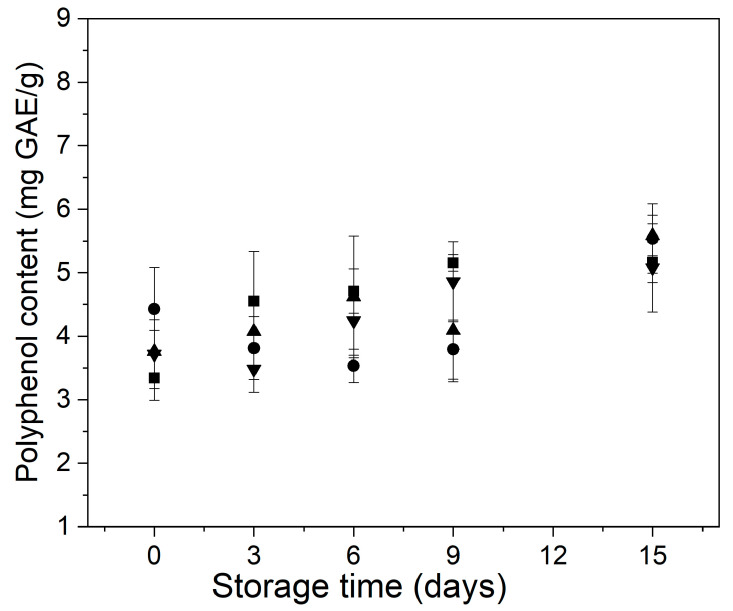
Behavior of the content of phenolic compounds of control and treated minimally processed bunching green onion during storage. Control (■); mild-heat treatment (●); edible coating (▲); mild-heat treatment+ edible coating (▼).

Figure 6 shows the behavior of the total polyphenol content of control and treated minimally processed bunching green onions during storage. At the initial time, MHT samples (~4.4 mg GAE/g) showed higher polyphenol content than the samples control (~3.3 mg GAE/g), EC (~3.8 mg GAE/g), and MHT+ EC (~3.7 mg GAE/g). This behavior was similar to those observed by Siddiq et al. [47] when applied 60 °C heat treatment on fresh-cut onion (*Allium cepa* L.) slices finding that the mild-heat treatment caused a significant increase in the amount of phenolic compounds, from 4.4 to 5.2 mg GAE/g. The polyphenol content of the control and treated samples did not follow a common pattern during storage. During the first 3 days of storage, the content of phenolic compounds of control samples increased significantly, and this trend continued until the end of storage. In the case of the MHT samples, the polyphenol content diminished from day 0 to day 6 and then increased towards day 15 of the storage. The samples EC and MHT + EC showed a similar polyphenol content at the initial time. Moreover, those samples exhibited a gradual increase in their polyphenol content from day 0 to day 6. At day 15 of storage, all samples had higher polyphenol concentrations than at the initial time. It has been reported that the fresh-cut onions may increase their polyphenol content during storage in response to stresses such as damage and microbial infection [47,55]. 

**Figure 7:**
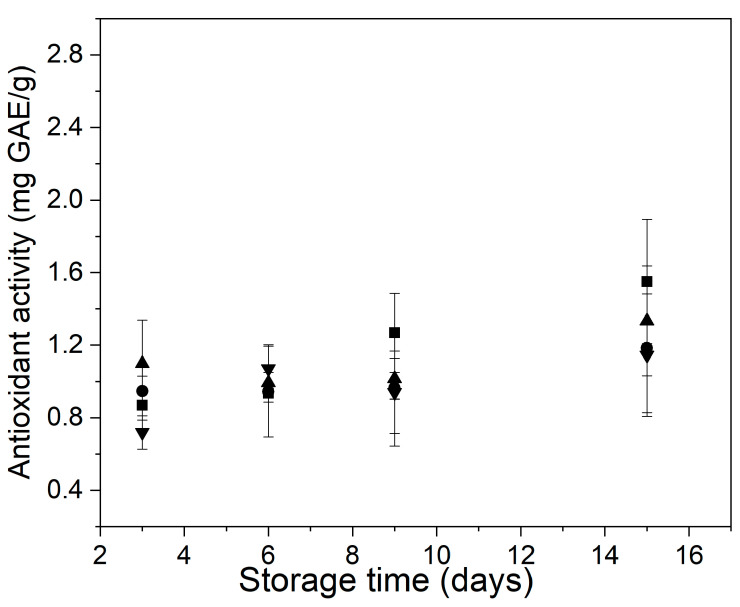
Behavior of the antioxidant activity of control and treated minimally processed bunching green onion during storage. Control (■); mild-heat treatment (●); edible coating (▲); mild-heat treatment+ edible coating (▼).

On the other hand, control and treated samples showed a similar antioxidant activity at the beginning of storage, which indicates that this parameter was not affected by the differences in the polyphenol content of the samples (Figure 7). In addition, all samples showed a slight increase in their antioxidant activity towards the end of the storage, mainly the samples control and EC. Siddiq et al., when evaluating the polyphenol amount and the antioxidant activity of fresh-cut onions treated with mild heat, found that the DPPH^●^ scavenging activity of the samples had no significant changes during 21 days of storage at 4 °C, except that the 70 °C treated sample showed higher antioxidant capacity at day 21 of storage. 

### 3.4. Microbial Count and Decay 

The counts of mesophilic aerobic and molds/yeast of control and treated minimally processed bunching green onion during storage are shown in Table 1. At the initial time, all samples showed a mesophilic aerobic count raging between 3.6 and 3.9 log CFU · g^−1^. During the storage, MHT and control samples exhibited a similar count of mesophilic aerobic bacteria, while the EC samples showed a slight increase in the microbial count regarding the control. This behavior suggests that the single application of MHT and EC did not cause a decrease in the mesophilic aerobic count. During the first 3 days of storage, the samples treated with the combination of MHT and EC showed a slight decrease in the mesophilic aerobic count, concerning the control and the samples EC and MHT. After this time, the mesophilic aerobic count in both control and treated samples gradually increased, reaching values less than 4.5 log CFU · g^−1^ at day 15 of storage.

At the initial time, the count of molds and yeasts of control samples was around 2.9 log CFU · g^−1^, while in all treated samples molds and yeast were not detected (Table 1). During the storage, the population of molds and yeasts increased in both control and treated samples, reaching counts less than 5.5 log CFU · g^−1^ at end of the storage. It has been reported that a maximum of 7 log CFU · g^−1^ for total counts is acceptable in minimally processed vegetable products [14]. Based on such criteria, all samples were safe to consume after 15 days of refrigerated storage.

**Figure 8:**
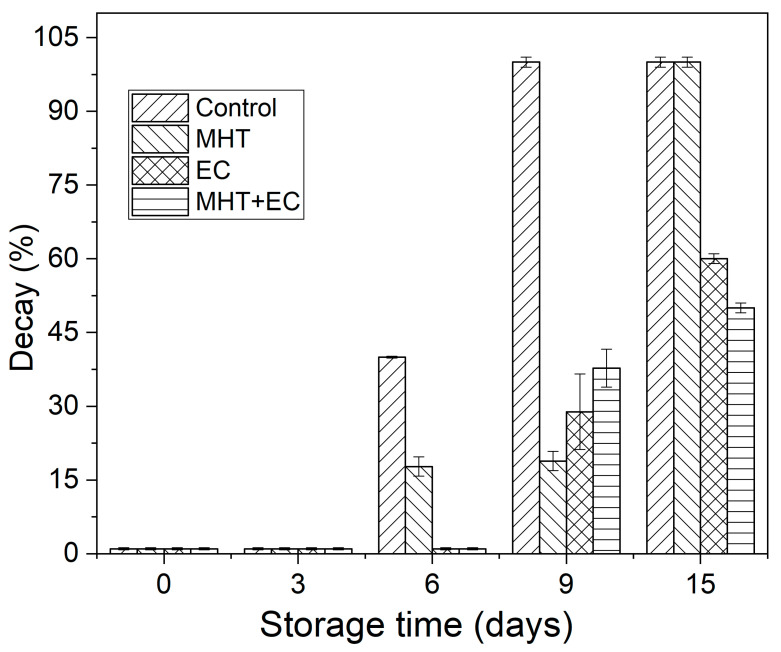
Incidence of decay in control and treated minimally processed bunching green onion during storage.

Figure 8 shows the incidence of decay in control and treated minimally processed bunching green onion during storage. In addition, images of the appearance of the samples are shown in Figure S1. Control and treated samples showed a low incidence of decay at the first 3 days of storage (Figure 8 and Figure S1). On day 6 of storage, the samples control and MHT showed some symptoms of decay (e.g., red discoloration) which increased until the end of storage, reaching a decay ratio of 100% at days 9 and 15, respectively. In the case of the samples EC and MHT + EC, the apparition of signs of decay was delayed until day 9 of storage. Changes in the appearance of green onions are one of the main causes of losses during marketing because consumers associate these changes in appearance with loss of quality. It has been reported that vegetables of the genus *Allium* can develop pink, red (onion), green (garlic), or blue discoloration as a consequence of cell rupture [50,55]. The chemistry of this discoloration is specific to the genus *Allium* and both the enzyme isoalliin and alliinase are required [56]. 

On the other hand, after day 6 of storage, all samples showed barely detectable signs of extension growth (telescoping) [12]. According to Cantwell et al., a growth of 5 mm was not sufficiently noticeable to make the minimally processed green onions unmarketable unless they also had other defects [12]. 

### 3.5. Consumer Assessment

**Figure 9:**
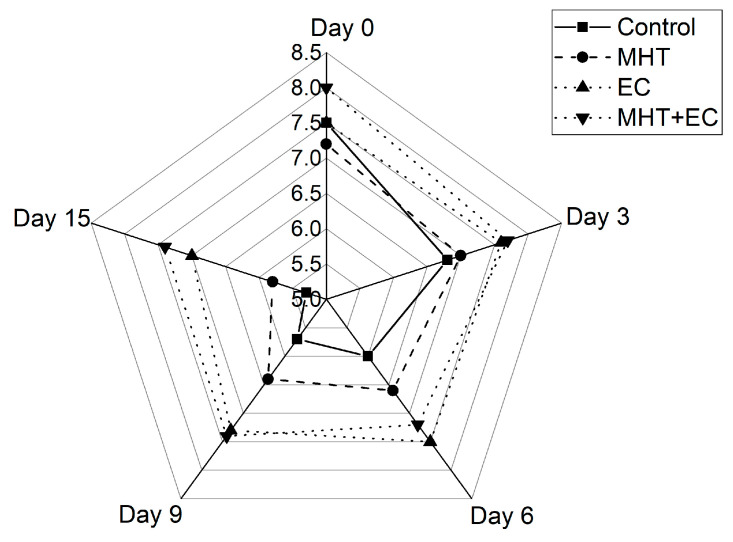
Radar chart representing mean scores of the overall visual quality of control and treated minimally processed green onions during storage.

Figure 9 shows the overall visual quality scores of control and treated minimally processed bunching green onions. At the initial time, all samples received overall visual quality scores ranging between 7.0 and 8.0 points, which indicates that the applied treatments would not compromise the consumer acceptance. From day 0 to day 3, the control samples showed a decrease in their overall visual quality scores of around 9.3%, while in the treated sample this percentage was below 5%. At the end of the storage, the samples control, MHT, EC and MHT + EC showed a decrease in their overall quality scores of around 30%, 19%, 6.7%, and 8.7%, respectively. It has been reported that a score of 6 is the limit of marketability in minimally processed green onion [14]. Based on such a criterion, only the samples EC and MHT + EC remained over the limit of usability (score 6) at day 15 of storage, which indicates that the application of edible coatings, single or in combination with MHT, maintained better overall visual quality along 15 days of storage. Those findings agreed with those reported by Zudaire [9], where the overall visual quality of untreated fresh-cut calçots decreased drastically in the first 3 days of storage, reaching scores less than the limit of usability, while that in the treated samples with sodium hypochlorite, peroxyacetic acid or mild-heat treatment maintained scores above 6 after 15 days of storage at 4 °C. Similar observations were also reported by Han et al. [54] and Hong et al. [14] to fresh-cut Welsh onions (*Allium fistulosum* L.) stored at 4 °C.

Overall, these results are suggesting that if a score of 6.0 for overall visual quality is defined as the limit of marketability, the shelf-life of untreated minimally processed green onions (~6 days), might be extended to less than 9 days with the application of MHT, and over 15 days with EC and MHT + EC.

## 4. Conclusions

Mild-heat treatment and alginate edible coatings proved to be useful and simple methods that can be applied individually or in combination to prevent the spoilage of minimally processed bunching green onions during refrigerated storage. These treatments did not affect the water loss and were able to delay firmness changes of the minimally processed bunching green onions during the storage. 

The treatments with alginate coating alone or applied after mild-heat treatment (55 °C for 60 s) were more effective for reducing the respiration rate and delayed the apparition of discoloration defects in the minimally processed bunching green onions during the storage. After 15 days of refrigerated storage, the best scores of overall visual quality were obtained when edible coatings were applied. These promising findings suggest that the combination of the hurdles studied in the current work could be considered further as an alternative means for maintaining the quality of minimally processed bunching green onions.

## Figures and Tables

**Table 1:** Behavior of the mesophilic aerobic and molds/yeasts grown on control and treated minimally processed bunching green onion during storage.

Microorganims	Samples	Day 0	Day 3	Day 6	Day 9	Day 15
Mesophilic Aerobic (Log CFU · g^−1^)	Control	3.72 ± 0.01 ^b^	3.78 ± 0.05 ^b^	3.94 ± 0.01 ^a^	4.02 ± 0.10 ^a^	4.16 ± 0.08 ^a^
	MHT	3.76 ± 0.01 ^b^	3.77 ± 0.01 ^b^	3.95 ± 0.05 ^a^	4.01 ± 0.07 ^a^	4.16 ± 0.04 ^a^
	EC	3.85 ± 0.02 ^a^	3.89 ± 0.01 ^a^	4.07 ± 0.01 ^a^	4.21 ± 0.10 ^a^	4.36 ± 0.01 ^a^
	MHT + EC	3.63 ± 0.02 ^c^	3.66 ± 0.01 ^c^	4.02 ± 0.11 ^a^	4.18 ± 0.10 ^a^	4.29 ± 0.08 ^a^
Molds and yeasts (Log CFU · g^−1^)	Control	2.87 ± 0.04 ^a^	3.79 ± 0.03 ^b^	3.97 ± 0.02 ^b^	4.10 ± 0.03 ^bc^	4.24 ± 0.10 ^b^
	MHT	nd ^b^	3.40 ± 0.02 ^c^	3.50 ± 0.05 ^d^	4.00 ± 0.04 ^c^	4.14 ± 0.01 ^b^
	EC	nd ^b^	4.65 ± 0.01 ^a^	4.83 ± 0.02 ^a^	5.20 ± 0.01 ^a^	5.32 ± 0.04 ^a^
	MHT + EC	nd ^b^	3.54 ± 0.06 ^c^	3.69 ± 0.04 ^c^	4.20 ± 0.01 ^b^	4.28 ± 0.01 ^b^

MHT: mild-heat treatments; EC: edible coating; MHT + EC: combination of mild-heat treatment and coating applications. nd: not detected. ^a,b,c,d^ Different letters in each column correspond to significant differences (*p* < 0.05).

## Data Availability

The data presented in this study are available on request from the corresponding author.

## Supplementary Materials

The following supporting information can be downloaded at: https://www.mdpi.com/article/10.3390/foods11050641/s1, Figure S1: Images of control and treated minimally processed bunching green onion stored at 4 °C for 15 days.

## References

[1] Grzegorzewska M., Badełek E., Szczech M., Kosson R., Wrzodak A., Kowalska B., Colelli G., Szwejda-Grzybowska J., Maciorowski R. (2022). The effect of hot water treatment on the storage ability improvement of fresh-cut Chinese cabbage. Sci. Hortic. (Amst.).

[2] Botondi R., Barone M., Grasso C. (2021). A Review into the Effectiveness of Ozone Technology for Improving the Safety and Preserving the Quality of Fresh-Cut Fruits and Vegetables. Foods.

[3] Testa R., Schifani G., Migliore G. (2021). Understanding Consumers’ Convenience Orientation. An Exploratory Study of Fresh-Cut Fruit in Italy. Sustainability.

[4] De Corato U. (2020). Improving the shelf-life and quality of fresh and minimally-processed fruits and vegetables for a modern food industry: A comprehensive critical review from the traditional technologies into the most promising advancements. Crit. Rev. Food Sci. Nutr..

[5] Lwin W., Srilaong V., Kanlayanarat S., Pongprasert N., Uthairatanakij A. (2013). Effects of Different Exposure Times of 1-MCP on the Quality of Bunching Onions (*Allium fistulosum*). Agric. Sci. J..

[6] Kurnia D., Ajiati D., Heliawati L., Sumiarsa D. (2021). Antioxidant Properties and Structure-Antioxidant Activity Relationship of *Allium* Species Leaves. Molecules.

[7] Țigu A.B., Moldovan C.S., Toma V.-A., Farcaș A.D., Moț A.C., Jurj A., Fischer-Fodor E., Mircea C., Pârvu M. (2021). Phytochemical Analysis and In Vitro Effects of *Allium fistulosum* L. and *Allium sativum* L. Extracts on Human Normal and Tumor Cell Lines: A Comparative Study. Molecules.

[8] Alvarez M.V., Moreira M.D.R., Roura S.I., Ayala-Zavala J.F., González-Aguilar G.A., Taylor T.M. (2015). 13—Using natural antimicrobials to enhance the safety and quality of fresh and processed fruits and vegetables: Types of antimicrobials. Woodhead Publishing Series in Food Science, Technology and Nutrition.

[9] Zudaire L., Viñas I., Abadias M., Simó J., Aguiló-Aguayo I. (2018). Efficacy of chlorine, peroxyacetic acid and mild-heat treatment on the reduction of natural microflora and maintenance of quality of fresh-cut calçots (*Allium cepa* L.). LWT.

[10] Zudaire L., Lafarga T., Viñas I., Abadias M., Brunton N., Aguiló-Aguayo I. (2019). Effect of Ultrasound Pre-Treatment on the Physical, Microbiological, and Antioxidant Properties of Calçots. Food Bioprocess Technol..

[11] Rozo G., Gómez D., Rozo C. (2016). Effect of an alginate edible film coating in the conservation of welsh onion (*Allium fistulosum* L.) | Efecto de una biopelícula de alginato en la conservación de cebolla larga (*Allium fistulosum* L.). Vitae.

[12] Cantwell M.I., Hong G., Suslow T.V. (2001). Heat treatments control extension growth and enhance microbial disinfection of minimally processed green onions. HortScience.

[13] Hong G., Peiser G., Cantwell M.I. (2000). Use of controlled atmospheres and heat treatment to maintain quality of intact and minimally processed green onions. Postharvest Biol. Technol..

[14] Hong S.I., Kim D. (2004). The effect of packaging treatment on the storage quality of minimally processed bunched onions. Int. J. Food Sci. Technol..

[15] Fallik E., Ilić Z., Pareek S. (2018). Hot Water Treatments. Novel Postharvest Treatments of Fresh Produce.

[16] Zhang L., Li S., Wang A., Li J., Zong W. (2017). Mild heat treatment inhibits the browning of fresh-cut *Agaricus bisporus* during cold storage. LWT—Food Sci. Technol..

[17] Mahajan P.V., Caleb O.J., Singh Z., Watkins C.B., Geyer M. (2014). Postharvest treatments of fresh produce. Philos. Trans. R. Soc. A Math. Phys. Eng. Sci..

[18] Moreira M.D.R., Ponce A., Ansorena R., Roura S.I. (2011). Effectiveness of Edible Coatings Combined with Mild Heat Shocks on Microbial Spoilage and Sensory Quality of Fresh Cut Broccoli (*Brassica oleracea* L.). J. Food Sci..

[19] Giannakourou M.C., Tsironi T.N. (2021). Application of Processing and Packaging Hurdles for Fresh-Cut Fruits and Vegetables Preservation. Foods.

[20] Miteluț A.C., Popa E.E., Drăghici M.C., Popescu P.A., Popa V.I., Bujor O.-C., Ion V.A., Popa M.E. (2021). Latest Developments in Edible Coatings on Minimally Processed Fruits and Vegetables: A Review. Foods.

[21] Khan M.R., Di Giuseppe F.A., Torrieri E., Sadiq M.B. (2021). Recent advances in biopolymeric antioxidant films and coatings for preservation of nutritional quality of minimally processed fruits and vegetables. Food Packag. Shelf Life.

[22] Medina Jaramillo C., Estevez Areco S., Goyanes S., López Córdoba A. (2019). Characterization of starches isolated from Colombian native potatoes and their application as novel edible coatings for wild Andean blueberries (*Vaccinium meridionale* Swartz). Polymers.

[23] Nair M.S., Tomar M., Punia S., Kukula-Koch W., Kumar M. (2020). Enhancing the functionality of chitosan- and alginate-based active edible coatings/films for the preservation of fruits and vegetables: A review. Int. J. Biol. Macromol..

[24] Zapata P.J., Castillo S., Valero D., Guillén F., Serrano M., Díaz-Mula H.M. (2010). The use of alginate as edible coating alone or in combination with essential oils maintained postharvest quality of tomato. Acta Hortic..

[25] Wüstenberg T., Wüstenberg T. (2015). General Overview of Food Hydrocolloids. Cellulose and Cellulose Derivatives in the Food industry Fundamentals and Applications.

[26] Medina-Jaramillo C., Quintero-Pimiento C., Gómez-Hoyos C., Zuluaga-Gallego R., López-Córdoba A. (2020). Alginate-Edible Coatings for Application on Wild Andean Blueberries (*Vaccinium meridionale* Swartz): Effect of the Addition of Nanofibrils Isolated from Cocoa By-Products. Polymers.

[27] Jayakody M.M., Vanniarachchy M.P.G., Wijesekara I. (2022). Seaweed derived alginate, agar, and carrageenan based edible coatings and films for the food industry: A review. J. Food Meas. Charact..

[28] Medina-Jaramillo C., Quintero-Pimiento C., Díaz-Díaz D., Goyanes S., López-Córdoba A. (2020). Improvement of Andean Blueberries Postharvest Preservation Using Carvacrol/Alginate-Edible Coatings. Polymers.

[29] Prakash A., Baskaran R., Vadivel V. (2020). Citral nanoemulsion incorporated edible coating to extend the shelf life of fresh cut pineapples. LWT.

[30] Robles-Sánchez R.M., Rojas-Graü M.A., Odriozola-Serrano I., González-Aguilar G., Martin-Belloso O. (2013). Influence of alginate-based edible coating as carrier of antibrowning agents on bioactive compounds and antioxidant activity in fresh-cut Kent mangoes. LWT—Food Sci. Technol..

[31] Poverenov E., Danino S., Horev B., Granit R., Vinokur Y., Rodov V. (2014). Layer-by-Layer Electrostatic Deposition of Edible Coating on Fresh Cut Melon Model: Anticipated and Unexpected Effects of Alginate–Chitosan Combination. Food Bioprocess Technol..

[32] Sarengaowa, Hu W., Jiang A., Xiu Z., Feng K. (2018). Effect of thyme oil–alginate-based coating on quality and microbial safety of fresh-cut apples. J. Sci. Food Agric..

[33] Ansorena M.R., Marcovich N.E., Roura S.I. (2011). Impact of edible coatings and mild heat shocks on quality of minimally processed broccoli (*Brassica oleracea* L.) during refrigerated storage. Postharvest Biol. Technol..

[34] Koh P.C., Noranizan M.A., Karim R., Nur Hanani Z.A., Lasik-Kurdyś M. (2018). Combination of alginate coating and repetitive pulsed light for shelf life extension of fresh-cut cantaloupe (*Cucumis melo* L.. reticulatus cv. Glamour). J. Food Process. Preserv..

[35] Ben-Fadhel Y., Cingolani M.C., Li L., Chazot G., Salmieri S., Horak C., Lacroix M. (2021). Effect of γ-irradiation and the use of combined treatments with edible bioactive coating on carrot preservation. Food Packag. Shelf Life.

[36] Piñeros-Hernandez D., Medina-Jaramillo C., López-Córdoba A., Goyanes S. (2017). Edible cassava starch films carrying rosemary antioxidant extracts for potential use as active food packaging. Food Hydrocoll..

[37] Singleton V.L., Orthofer R., Lamuela-Raventos R.M., Lester Packer L. (1999). Analysis of total phenols and other oxidation substrates and antioxidants by means of Folin-Ciocalteu reagent. Methods in Enzymology (Oxidants and Antioxidants, Part A).

[38] Estevez-Areco S., Guz L., Famá L., Candal R., Goyanes S. (2019). Bioactive starch nanocomposite films with antioxidant activity and enhanced mechanical properties obtained by extrusion followed by thermo-compression. Food Hydrocoll..

[39] Brand-Williams W., Cuvelier M.E., Berset C. (1995). Use of a free radical method to evaluate antioxidant activity. LWT—Food Sci. Technol..

[40] (2013). Microbiology of the Food Chain—Horizontal Method for the Enumeration of Microorganisms—Part 1: Colony Count at 30 C by the Pour Plate Technique.

[41] (2008). Microbiology of Food and Animal Feeding Stuffs—Horizontal Method for the Enumeration of Yeasts and Moulds—Part 2: Colony Count Technique in Products with Water Activity Less Than or Equal to 0.95.

[42] Moskowitz H.R., Beckley J.H., Resurreccion A.V.A., Moskowitz H.R., Beckley J.H., Resurreccion A.V.A. (2012). What types of tests do sensory researchers do to measure sensory response to the product? and …why do they do them?. Sensory and Consumer Research in Food Product Design and Development.

[43] Alharaty G., Ramaswamy H.S. (2020). The Effect of Sodium Alginate-Calcium Chloride Coating on the Quality Parameters and Shelf Life of Strawberry Cut Fruits. J. Compos. Sci..

[44] Xylia P., Chrysargyris A., Tzortzakis N. (2021). The Combined and Single Effect of Marjoram Essential Oil, Ascorbic Acid, and Chitosan on Fresh-Cut Lettuce Preservation. Foods.

[45] Romanazzi G., Feliziani E., Sivakumar D. (2018). Chitosan, a Biopolymer With Triple Action on Postharvest Decay of Fruit and Vegetables: Eliciting, Antimicrobial and Film-Forming Properties. Front. Microbiol..

[46] Senturk Parreidt T., Müller K., Schmid M. (2018). Alginate-Based Edible Films and Coatings for Food Packaging Applications. Foods.

[47] Siddiq M., Roidoung S., Sogi D.S., Dolan K.D. (2013). Total phenolics, antioxidant properties and quality of fresh-cut onions (*Allium cepa* L.) treated with mild-heat. Food Chem..

[48] Mcglynn W. (2003). The Importance of Food pH in Commercial Canning Operations. Food Technology Fact Sheet.

[49] Zudaire L., Viñas I., Abadias M., Lafarga T., Bobo G., Simó J., Aguiló-Aguayo I. (2019). Effects of long-term controlled atmosphere storage, minimal processing, and packaging on quality attributes of calçots (*Allium cepa* L.). Food Sci. Technol. Int..

[50] Toivonen P.M.A., Brummell D.A. (2008). Biochemical bases of appearance and texture changes in fresh-cut fruit and vegetables. Postharvest Biol. Technol..

[51] Andrés-Bello A., Barreto-Palacios V., García-Segovia P., Mir-Bel J., Martínez-Monzó J. (2013). Effect of pH on Color and Texture of Food Products. Food Eng. Rev..

[52] Kumar N., Pratibha, Neeraj, Ojha A., Upadhyay A., Singh R., Kumar S. (2021). Effect of active chitosan-pullulan composite edible coating enrich with pomegranate peel extract on the storage quality of green bell pepper. LWT.

[53] Kocira A., Kozłowicz K., Panasiewicz K., Staniak M., Szpunar-Krok E., Hortyńska P. (2021). Polysaccharides as Edible Films and Coatings: Characteristics and Influence on Fruit and Vegetable Quality—A Review. Agronomy.

[54] Han C., Ji Y., Li M., Li X., Jin P., Zheng Y. (2016). Influence of wounding intensity and storage temperature on quality and antioxidant activity of fresh-cut Welsh onions. Sci. Hortic. (Amst.).

[55] Howard L.R., Yoo K.S., Pike L.M., Miller G.H. (1994). Quality Changes in Diced Onions Stored in Film Packages. J. Food Sci..

[56] Kubec R., Hrbáčová M., Musah R.A., Velíšek J. (2004). *Allium* Discoloration:  Precursors Involved in Onion Pinking and Garlic Greening. J. Agric. Food Chem..

