# Supplementary material for: Single and Combined Effect of Mild-Heat Treatment and Alginate Coatings on Quality Preservation of Minimally Processed Bunching Green Onions
Source: Foods. 2022 Feb 23;11(5):641. doi: 10.3390/foods11050641 (PMC8909205; doi:10.3390/foods11050641)
Supplement: Supplementary file 1 [file foods-11-00641-s001.zip › foods-1591580-supplementary.pdf]

foods

Supplementary material

Single and combined effect of mild-heat treatment and alginate coatings on quality preservation of minimally processed bunching green onions

Carolina Medina-Jaramillo, Karen Usgame-Fagua, Nelson Franco-González and Alex López Córdoba *

Grupo de Investigación en Bioeconomía y Sostenibilidad Agroalimentaria. Universidad Pedagógica y Tecnológica de Colombia. Facultad Seccional Duitama. Escuela de Administración de Empresas Agropecuarias. Carrera18 con Calle 22 Duitama, Boyacá, Colombia.

* Correspondence: alex.lopez01@uptc.edu.co; Tel.: +57 8 7604100.

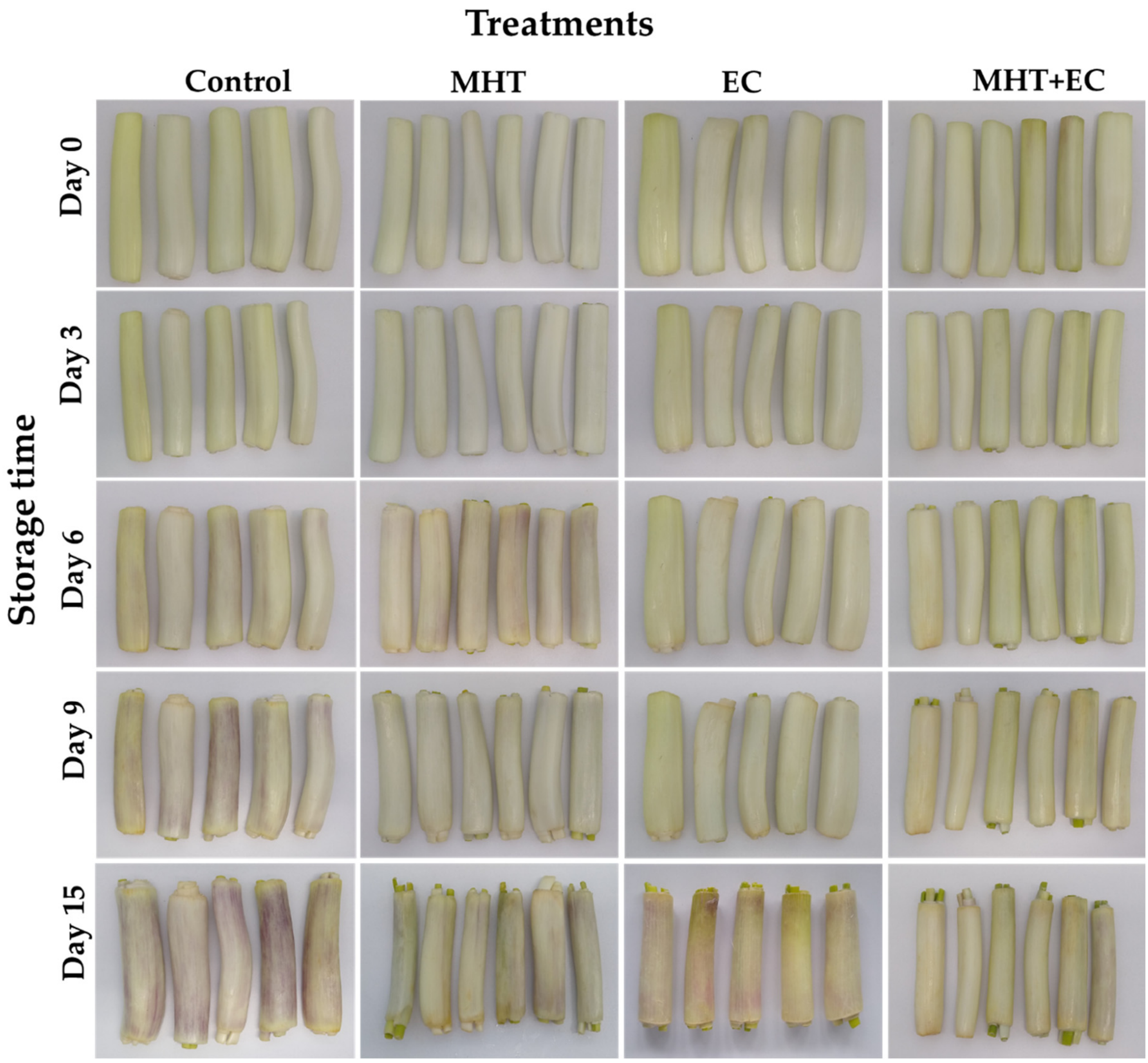

Figure S1. Images of control and treated minimally processed bunching green onion stored at 4ºC for 15 days
